# Has the Standard Cognitive Reflection Test Become a Victim of Its Own Success?

**DOI:** 10.5709/acp-0193-5

**Published:** 2016-09-30

**Authors:** Matthew Haigh

**Affiliations:** Department of Psychology, Northumbria University, UK

**Keywords:** Cognitive Reflection Test,, CRT, bat and ball problem, validity, test security

## Abstract

The Cognitive Reflection Test (CRT) is a hugely influential problem solving task that measures individual differences in the propensity to reflect on and override intuitive (but incorrect) solutions. The validity of this three-item measure depends on participants being naïve to its materials and objectives. Evidence from 142 volunteers recruited online suggests this is often not the case. Over half of the sample had previously seen at least one of the problems, predominantly through research participation or the media. These participants produced substantially higher CRT scores than those without prior exposure (2.36 vs. 1.48), with the majority scoring at ceiling level. Participants that had previously seen a specific problem (e.g., the bat and ball problem) nearly always solved that problem correctly. These data suggest the CRT may have been widely invalidated. As a minimum, researchers must control for prior exposure to the three problems and begin to consider alternative, extended measures of cognitive reflection.

## Introduction

 Dual process models of human cognition typically make a distinction between fast and autonomous Type 1 thinking and slower, consciously controlled Type 2 thinking ( Evans, 2008). The advantage of Type 1 thinking is that it produces quick and approximate solutions to a given problem at little computational expense. However, while this system often provides good enough responses, it is susceptible to being misled. One of the main roles of Type 2 thinking is to reflect on and override such intuitive, but incorrect responses ( Evans & Stanovich, 2013). 

 People vary in their propensity to spontaneously engage in this type of reflective thought and these individual differences are associated with a variety of everyday consequences (Pennycook, Fugelsang, & Koehler, 2015). Pennycook et al. (2015) concluded that more reflective thinkers tend to be more rational, more sceptical, less religious, and hold fewer epistemically suspect beliefs. By far the most popular performance measure of individual differences in this propensity is known as the Cognitive Reflection Test (CRT; Frederick, 2005). The standard version of the CRT is made up of three problems, including the well-known bat and ball problem: 

A bat and a ball cost $1.10 in total. The bat costs $1.00 more than the ball.

## How much does the ball cost? (in cents) _________

 The three problems all lure people towards an intuitive answer, driven by Type 1 processing. In the bat and ball problem, the intuitive answer that quickly comes to mind is 10 cents. However, this is incorrect. Those that spontaneously reflect on their response (using Type 2 processes) quickly realise that the correct answer is actually 5 cents. Participants can score a minimum of zero and a maximum of three on this measure. Across a combined sample of 3,428 participants from 35 separate studies, Frederick (2005) reported a mean score of 1.24 correct answers (sample means ranged from 0.57 to 2.18), suggesting that this measure is sensitive enough to capture individual differences and does not suffer unduly from ceiling effects (with only 17% of participants answering all three problems correctly). Importantly, variation on this measure is associated with theoretically relevant outcomes (Pennycook et al., 2015) and taps into cognitive processes that are independent of intelligence and executive functioning (Toplak, West, & Stanovich, 2011). 

 The CRT has been hugely influential. The original paper (Frederick, 2005) is cited over 1,700 times1, while the test has received extensive media and social media coverage. For example, it is featured in bestselling popular psychology books such as Daniel Kahneman’s (2011) *Thinking Fast and Slow*. It has also been widely shared on social media, has been featured in the press (e.g., *Business Insider* and *the New York Times*), and is a staple component of introductory psychology courses (Thomson & Oppenheimer, 2016). 

 The validity of the CRT depends on participants being blind to its objectives, but this required level of naivety is threatened by widespread publication of the test materials. Once the logic behind this measure is known (or the solutions to any of the three problems are known), then the task is essentially invalidated as measure of cognitive reflection for that participant. Both the American Psychological Association (APA, 2016) and the British Psychological Society (BPS, 2016) warn that widespread public disclosure of the objectives or materials of a psychological test can cause irreparable harm to its validity. This is of particular relevance to the CRT for three reasons. First, the nature of the test requires that participants are naïve to its objectives. If participants are aware that the three problems are "trick questions", they will be less likely to trust their instincts and be more likely to engage a critical, Type 2 style of thinking (Chandler, Mueller, & Paolacci, 2014). Second, the test only consists of three items, each of which has a single correct answer, meaning that some people may simply memorise that correct answer. Third, the populations typically sampled by psychologists (i.e., psychology undergraduates and participants recruited through online participant pools) are precisely the groups that are most likely to have previously come across the CRT. 

 In this paper, it is argued that the standard version of the CRT is becoming a victim of its own success. Individual variation in the CRT reveals the capacity for reflecting on Type 1 responses, but as this measure becomes increasingly popular among researchers and is more widely publicised by the media, this variation may be polluted by prior exposure to the test materials. Toplak, West, and Stanovich (2014) noted the potential for future complications with the CRT as the three items become increasingly well known, while Thomson and Oppenheimer (2016) presented evidence that most participants recruited using the Amazon Mechanical Turk (MTurk) had previously encountered at least one problem from the CRT, and that these participants significantly outperformed those with no prior experience (mean scores of 1.90 vs. 1.29). Similarly, Chandler et al. (2014) tested participants recruited using MTurk and found a strong positive correlation between the extent of prior participation in online studies and performance on CRT (*r* = .79) but no such correlation for a novel version of the CRT, in which the standard wording of the problems was changed (*r* = .04). 

 These data suggest that the CRT is at risk of becoming an invalid measure of cognitive reflection, yet researchers habitually fail to control for prior exposure. In the experiment reported below, participants completed the CRT and were then asked about their prior exposure to each of the three problems. The first aim was to replicate findings described above using a more general online sample. Both Thomson and Oppenheimer (2016) and Chandler et al. (2014) recruited participants through MTurk, which has recently received attention for having much smaller and less diverse pool of participants than previously thought (Stewart et al., 2015), thus increasing the likelihood of prior exposure. Therefore we recruited participants through a variety of different online platforms (detailed in the Method section) to try and access a wider population of potential participants and see if the effect extends beyond the limited MTurk population. The second aim was to build on these prior findings by examining the extent to which exposure to any given CRT problem influences performance on that specific problem. Finally, the third aim was to identify the source of any such exposure (e.g., in previous research or in the media). 

 It is hypothesised that participants who report prior exposure to at least one problem from this measure will produce substantially more correct answers than those without prior exposure (replicating the results of Thomson & Oppenheimer, 2016). However, not all of these participants will have seen all of the problems. The bat and ball problem, for instance, is thought to be much more well-known than the other two problems (Toplak et al., 2014). A more specific prediction is that participants who have previously seen a specific problem (e.g., the bat and ball problem) will outperform the remaining participants on that specific problem, with a level of performance that is close to ceiling. To identify the source of prior exposure, participants were also asked to disclose where they had previously come across the materials (e.g., in previous research or in the media). These data will allow for an assessment of the extent to which the three CRT items have been invalidated by prior exposure, while also identifying the sources of this exposure in an effort to raise awareness around issues of test security (cf. APA, 2016; BPS 2016). 

## Method

### Design and Participants

The primary aim of this study was to compare performance on the CRT between those participants that reported prior exposure to any of the three problems and those with no prior exposure. Therefore the a priori sample size was calculated for a two-tailed between-subjects *t*-test with an anticipated effect size of *d* = 0.5 (medium), a desired power level of .8 and a probability level of .05. Based on these assumptions, the a priori minimum sample size was calculated to be 128.

Volunteers responded to online adverts seeking participants to take part in a short problem solving task. The adverts were posted to social media sites (Reddit, Facebook, Twitter) and online participant pools (Call for Participants [www.callforparticipants.com] and Prolific Academic [www.prolific.ac]). Posts on social media sites were only made to dedicated research participation forums or groups (e.g., Reddit Participants, Reddit Sample Size, and the Psychology Studies Twitter feed). Participants who completed the study via Prolific Academic (*n* = 44) were paid £0.5, while the remaining participants were not offered an incentive. A total of 142 volunteers completed the task (65 male, 72 female, 5 preferred not to disclose their gender; *M*_age_ = 28 years). A further 23 volunteers consented to take part but provided no data, so their responses were excluded.

### Materials and Procedure

 Participants were first asked to complete the standard CRT (Frederick, 2005). After completing the CRT, they were asked, “Have you ever come across any of these three problems before today?”, with the response options “Yes”, “No”, and “Don’t know.” The primary aim of this study was to compare performance on the CRT between those that had prior exposure to this measure (those that answered “Yes”) and those with no prior exposure (those that answered “No” or “Don’t Know”). Those participants that answered positively were presented with three additional questions. The first asked them to select which of the three items they had seen previously (“Which of the three problems have you come across before today? [Select all that apply]”), with the options, “Problem 1 - BAT AND BALL”, “Problem 2 - WIDGETS”, “Problem 3 - LILY PADS”). The second asked if they believed they had previously seen the correct answer to these problems, with the response options “Yes”, “No”, and “Don’t know” (the correct answers were not given to participants). The third asked participants to describe where they had previously seen the items (with responses typed into a text entry field). 

## Results

The overall mean score on the CRT was 1.93 correct answers (*SD* = 1.17). A total of 73 participants (51.4%) reported that they had previously been exposed to at least one of the problems from the CRT. Of these participants, 33.3% had seen the problems in previous research studies, 30.6% had seen them in popular media, which included books, websites and social media, 22.2% had seen them in school or university, while 18.1% either did not know or did not answer2. Of the remaining participants, 56 (39.4%) stated that they had not come across any of the problems before, while 13 (9.2%) did not know if they had come across the problems before. The latter two groups were combined in the analyses reported below.

 The mean score of those with prior exposure to any problem from the CRT (*M* = 2.36, *SD* = 0.96) was significantly higher than the mean score of those with no prior exposure to the CRT (*M* = 1.48, *SD* = 1.21; *t*[140] = 4.802, *p* < .001, *d* = 0.81). The group that had previously been exposed to the CRT produced, on average, 0.88 more correct answers than the group with no prior exposure. This was a large effect (d > 0.80; Cohen, 1977). Table 1 shows that the majority of participants in the prior exposure group obtained the maximum possible score. Within the prior exposure group the specific source of exposure (e.g., previous research, media, school or university etc.) had no effect on CRT mean scores, F(3, 71) = 1.54, *p* = .213. 

**Table 1.** Percentage of Participants Scoring 0, 1, 2, or 3 on the CRT Stratified by Prior Exposure to Any of the Three Problems

		Percentage of participants scoring 0, 1, 2, or 3			
	Mean CRT	0	1	2	3
Prior Exposure (*n* = 73)	2.36	8.2%	9.6%	20.5%	61.6%
No Prior Exposure (*n* = 69)	1.48	30.4%	20.3%	20.3%	29.0%
Overall (*N* = 142)	1.93	19.0%	14.8%	20.4%	45.8%

*Note*. CRT = Cognitive Reflection Test.

Some of those who had previous exposure to the CRT also believed they had previously seen the correct answer to at least one of the questions (*n* = 34). These participants scored higher on the CRT (*M* = 2.59, *SD* = 0.82) than those who had previous exposure but had not previously seen any correct answers (*M* = 2.13, *SD* = 1.04; *t*[70] = 2.05, *p* = .045, *d* = 0.49) and higher than those with no previous exposure to the CRT (*M* = 1.48, *SD* = 1.21; *t*[101] = 4.83, *p* < .001, *d* = 1.07).

The analyses above show that exposure to at least one CRT problem increases scores on the test as a whole. One reason for this may be due to participants scoring close to ceiling level on those specific problems that they have previously been exposed to. If they remember a specific problem, they may also remember that the problem is a trick question (or even remember the correct answer, if known). It is therefore predicted that responses to previously seen problems will nearly always be correct. To investigate this, participants that had been previously exposed to the CRT were also asked which of the three problems they had seen previously. An individual item analysis was conducted to determine whether the effect of prior exposure to a specific item influenced performance on that specific item. It is conceivable that one or more items may be relatively immune from the effect of prior exposure (e.g., if the correct solution to a given problem is easily forgettable, or if a given problem seems less like a trick question than the others). The individual item analysis presented in Table 2 shows that none of the items were immune to the effects of prior exposure. Prior exposure to any one of the three problems was associated with a greater percentage of participants providing a correct answer to that specific problem. Indeed, the majority of participants with prior exposure to a specific problem solved that problem correctly. This is just one way in which prior exposure may influence total score on the CRT. It is also likely that knowing at least one problem is a trick question leads participants to treat other (previously unseen) problems as trick questions, hence improving performance on these previously unseen items. However, this possibility could not be tested directly with the data available, as the majority of participants had previously seen either all three of the CRT problems or none of the CRT problems.

**Table 2.** Percentage of Participants that Provided a Correct Solution to Each of the Three Problems, Stratified by Previous Exposure to that Specific Problem

	% correct responses to each problem		
	Prior exposure	No prior exposure	Chi-square
Problem 1: Bat and Ball	73.8% (*n* = 61)	40.7% (*n* = 81)	χ^2^(1, *N* = 142) = 15.33, *p* < .001
Problem 2: Widgets	87.2% (*n* = 47)	53.7% (*n* = 95)	χ^2^(1, *N* = 142) = 15.51, *p* < .001
Problem 3: Lily Pads	86.5% (*n* = 52)	63.3% (*n* = 90)	χ^2^(1, *N* = 142) = 8.77, *p* < .003

## Discussion

 The CRT is a hugely influential measure of individual differences in the propensity to override Type 1 intuitions. It is uniquely associated with performance on a number of heuristics and biases tasks (Toplak et al., 2014) and with a variety of everyday consequences (Pennycook et al., 2015). However, the data presented here and elsewhere (Chandler et al., 2014 ; Thomson & Oppenheimer, 2016) suggest that it is becoming a victim of its own success. 

 Over half of the participants in this study (51.4%) had previously seen at least one problem from the CRT. Most of these had encountered the materials through previous research participation, in the popular media, or in school or university. This group scored very highly on the CRT, with an average of 2.36 correct answers (from a possible maximum of three). They significantly outperformed participants with no prior exposure to this measure (*M* = 1.48) in addition to outperforming the mean score of 1.24, which Frederick (2005) calculated from a sample of over 3,000 participants. Indeed, the majority of participants who reported prior exposure to at least one item performed at ceiling level. Analysis based on exposure to specific test items revealed that nearly all participants with prior exposure to a specific problem went on to solve that problem correctly. 

These findings suggest that performance on the CRT is not only influenced by individual differences in the propensity for cognitive reflection, but is also heavily influenced by individual differences in prior exposure. Those who had previously seen one or more of the problems produced higher scores on this measure. Therefore, the CRT may no longer be a valid measure among this group of participants3. Researchers habitually fail to ask about or control for prior exposure to the three problems, but given the results outlined above it is recommended that from now on those using the standard CRT (or any of its variants) should explicitly ask participants about prior exposure. The most conservative method of dealing with prior exposure is to exclude those participants that have previously seen any of the problems. This is particularly recommended when only a small proportion of a sample has previously been exposed to the CRT. However, when a large proportion of the sample has previously been exposed, as is typically the case following online recruitment, a more pragmatic approach could be considered. Rather than excluding what could be over half of the sample, an alternative approach is to statistically control for this issue by treating exposure as a between subjects factor. Any differences between these groups should then be clearly acknowledged.

 Given the growing popularity of the CRT, the problem of exposure is only likely to get worse. Fortunately, there is a simple solution. Chandler et al. (2014) showed that performance on a novel version of the CRT did not correlate with prior experience of online studies; therefore changes to the surface content of these problems may be sufficient to negate the effects of prior exposure. Indeed, there are now several alternative measures, including extended four-item and seven-item versions of the CRT (Toplak et al., 2014), the four-item CRT-2 ( Thomson & Oppenheimer, 2016) and the six-item CRT-Long (Primi, Morsanyi, Chiesi, Donati, & Hamilton, 2015). These measures offer validated alternatives but differ in the degree to which they overcome the problems described above. For example, the CRT-Long and the seven-item version of the CRT developed by Toplak et al. (2014) still contain the three original problems used by Frederick (2005) , which may have been widely invalidated. However, the four-item version developed by Toplak et al. (2014) contains none of the original items, nor does the CRT-2 (Thomson & Oppenheimer, 2016). 

 A limitation of all the new measures described above is that they are still relatively short (the longest is made up of seven problems), and therefore the individual items risk invalidation if circulated widely. In addition, all of the new problems used in these tests maintain the appearance of a trick question (i.e., where the solution seems too obvious to be correct). Many research participants are now aware of the logic behind this type of question, possibly encouraging them to engage a more critical, Type 2 style of thinking. Nevertheless, these measures provide an improvement on the original CRT, but work is still required to create longer measures with problems that avoid the appearance of being a trick question. One possible avenue for achieving this goal is to make use of belief bias syllogisms, as these problems can lead people to endorse intuitively believable, yet logically invalid conclusions. These problems differ in surface structure from the standard CRT problems but strongly correlate with the CRT and predict the same cognitive outcome measures (Thomson & Oppenheimer, 2016). 

The issue of test security is one that researchers should be aware of. Short, well publicised measures such as the CRT are particularly vulnerable to invalidation and this problem is particularly acute amongst populations that regularly take part in psychology research (e.g., online and undergraduate participant pool members) and those who take an interest in popular psychology. Indeed, these groups are far from independent, so it should not be surprising that such a high percentage have come across this measure before. Researchers who use the CRT or any of its variants should be wary of this limitation and take measures to control for it.
